# Combined Effect of Metals, PFAS, Phthalates, and Plasticizers on Cardiovascular Disease Risk

**DOI:** 10.3390/toxics13060476

**Published:** 2025-06-05

**Authors:** Doreen Jehu-Appiah, Emmanuel Obeng-Gyasi

**Affiliations:** 1 Department of Built Environment, North Carolina A&T State University, Greensboro, NC 27411, USA; 2 Environmental Health and Disease Laboratory, North Carolina A&T State University, Greensboro, NC 27411, USA

**Keywords:** chemical mixtures, cardiovascular biomarkers, NHANES, Bayesian Kernel Machine Regression, environmental exposure

## Abstract

This study assessed the relationship between environmental chemical mixtures—including metals, per- and polyfluoroalkyl substances (PFAS), phthalates, and plasticizers—and key cardiovascular health markers using data from the 2013–2014 National Health and Nutrition Examination Survey (NHANES). The combined effects of these pollutants on cardiovascular markers were evaluated using Bayesian Kernel Machine Regression (BKMR), a flexible, non-parametric modeling approach that accommodates nonlinear and interactive relationships among exposures. BKMR was applied to assess both the joint and individual associations of the chemical mixture with systolic blood pressure (SBP), high-density lipoprotein (HDL), low-density lipoprotein (LDL), diastolic blood pressure (DBP), total cholesterol, and triglycerides. As part of the BKMR analysis, posterior inclusion probabilities (PIPs) were estimated to identify the relative importance of each exposure within the mixture. These results highlighted phthalates as major contributors to LDL, SBP, total cholesterol, HDL, and triglycerides while plasticizers were associated with LDL, SBP, HDL, and triglycerides. Metals and PFAS were most strongly linked to LDL, DBP, total cholesterol, and SBP. The overall mixture effect indicated that cumulative exposures were associated with lower LDL and SBP and elevated DBP, suggesting an increased cardiovascular risk. Triglycerides exhibited a complex quantile-dependent trend, with higher exposures associated with reduced levels. These findings underscore the importance of mixture-based risk assessments that reflect real-world exposure scenarios.

## 1. Introduction

Exposure to harmful chemicals, including metals, per- and polyfluoroalkyl substances (PFAS), phthalates, and plasticizers, is known to contribute to adverse health outcomes, particularly cardiovascular disease (CVD) [1,2]. These pollutants rarely occur in isolation; instead, they exist as mixtures that interact in complex ways, potentially amplifying or mitigating their health effects [3]. Understanding these exposure patterns is critical to identifying pollutant sources, pathways of exposure, and populations most at risk, ultimately enabling targeted interventions and more effective public health policies [4].

Among these pollutants, specific groups stand out for their strong associations with cardiovascular health risks. Metals such as lead and cadmium have long been associated with increased risks of hypertension and cardiovascular events [5,6]. PFAS, used in numerous consumer products, have been linked to cholesterol changes and vascular dysfunction [7]. Phthalates and plasticizers, common in plastics and personal care products, disrupt endocrine functions and may drive inflammation and oxidative stress, both key factors in CVD development [8,9]. Biomonitoring studies confirm that humans are exposed to many environmental chemicals across the life span, often simultaneously [10]. However, understanding the combined effects of these exposures remains a major challenge. This challenge arises from the complexity of analyzing chemical mixtures and their nonlinear interactions. Traditional methods such as Principal Component Analysis (PCA), multiple linear regression (MLR), and cluster analysis often fall short in capturing these complexities. For instance, PCA is limited by its reliance on linear assumptions and its inability to handle interactions between variables effectively [11]. Similarly, MLR struggles to address correlated exposures and nonlinear relationships, which are common in environmental data [12]. Cluster analysis, while useful for grouping similar exposures, lacks the capacity to model the effects of mixtures on health outcomes [13]. These limitations highlight the need for flexible methods like Bayesian Kernel Machine Regression (BKMR), a flexible, semi-parametric modeling approach that estimates nonlinear and interactive effects of multiple, potentially correlated exposures on an outcome by combining kernel machine methods with Bayesian inference.

To address that gap, this study examines the connections between chemical exposures using BKMR. BKMR analyzes the combined and nonlinear effects of chemical mixtures on health outcomes, helping to better understand how these exposures interact [14]. This method enables a deeper investigation into the complex relationships between environmental pollutants, their impact on cardiovascular health, and opportunities for creating targeted interventions

Biomarkers provide a non-invasive and reliable method for assessing internal exposure to environmental pollutants. These biomarkers are useful for measuring the body’s burden of harmful chemicals, including metals, phthalates, and plasticizers [15]. By analyzing biological samples, researchers can identify specific pollutants that individuals are exposed to and assess how these exposures may impact health outcomes.

A key resource for biomarker data is the National Health and Nutrition Examination Survey (NHANES) dataset, which includes detailed measurements of biomarkers of various environmental chemicals, such as cadmium, lead, and phthalates. NHANES data is widely used due to its representation of a large, diverse population, allowing for a meaningful analysis of exposure trends and associated health risks across demographic groups.

By examining biomarkers from NHANES and similar studies, researchers can identify populations at a greater risk of exposure, such as individuals living near industrial areas or those with limited access to clean water. This knowledge not only enhances our understanding of exposure risks but also informs public health strategies and regulatory policies aimed at minimizing exposure to harmful environmental pollutants [16].

Analyzing environmental pollutant mixtures is inherently challenging due to the higher-dimensional nature of the data when multiple pollutants are assessed simultaneously. This complexity makes it difficult to determine which specific pollutants contribute to adverse health outcomes. Many pollutants are correlated because they often share common sources, such as industrial emissions or consumer products, making it complicated to disentangle their individual and combined effects.

Recognizing these limitations, advanced approaches such as Bayesian methods are increasingly being explored [17,18].

This study leverages advanced statistical techniques to assess the relationships between complex pollutant mixtures and CVD risk. We employ BKMR to model the nonlinear, interactive effects of environmental chemical mixtures—such as cadmium, lead, and phthalates—on cardiovascular disease risk using biomarker data from the NHANES 2013–2014 dataset.

## 2. Materials and Methods

### 2.1. Data Source

This study utilized biomonitoring data from the NHANES 2013–2014 to examine the associations between environmental pollutant mixtures and CVD risk. The dataset includes measurements of urinary and serum biomarkers for various pollutants, including metals, PFAS, phthalates, and plasticizers—exposures that have been linked to adverse cardiovascular outcomes in previous research. The toxic exposures were analyzed in biological samples using standardized methods [19]. Briefly, urinary metal concentrations were measured using inductively coupled plasma mass spectrometry (ICP-MS; ELAN® DRC II, PerkinElmer SCIEX, Concord, ON, Canada) following a simple dilution step, allowing for sensitive and specific detection of multiple elemental isotopes. Serum concentrations of PFAS compounds were quantified using solid-phase extraction followed by high-performance liquid chromatography–tandem mass spectrometry (HPLC-MS/MS; Agilent 1200, Agilent Technologies Inc., Santa Clara, CA, USA; API 5500, AB Sciex, Concord, ON, Canada). Isomer-specific concentrations of PFOA and PFOS were summed to estimate total exposure. Urinary phthalate and plasticizer metabolites were assessed via HPLC-ESI-MS/MS (Surveyor HPLC and TSQ Quantum Ultra, Thermo Fisher Scientific, San Jose, CA, USA) following enzymatic deconjugation and online solid-phase extraction, with isotopically labeled internal standards (Cambridge Isotope Laboratories, Tewksbury, MA, USA) were used to ensure assay precision.

While NHANES includes sampling weights to allow for nationally representative estimates, we did not apply weights in this analysis. Our primary goal was to assess exposure–response relationships using a non-parametric BKMR framework, which focuses on conditional associations rather than population-level prevalence. Similar to prior applications of BKMR to NHANES data [14], we analyzed the biomarker subsample as is, with the understanding that results may not be generalizable to the full U.S. population. Future work may explore Bayesian modeling frameworks that explicitly incorporate a complex survey design.

### 2.2. Data Preprocessing

In this study, we addressed missing data using multiple imputation via the Amelia package in R, which applies an expectation-maximization with bootstrapping (EMB) algorithm. This method generates multiple plausible versions of the dataset by drawing imputed values from the posterior distribution, capturing both the central tendency and the uncertainty inherent in the missing data. We created ten such imputed datasets, each preserving the original distributional structure and inter-variable relationships to support unbiased and efficient parameter estimation.

To address missing data in the exposure variables, we performed multiple imputation using the Amelia package, generating 10 imputed datasets. Given the high computational demands of the BKMR framework when modeling high-dimensional mixtures using repeated Markov Chain Monte Carlo (MCMC) sampling, we employed an average imputation approach. Specifically, we averaged the imputed values across the 10 datasets to construct a single analysis dataset. This approach has been used in prior studies of complex models, including BKMR and machine learning applications, where full multiply-imputed analysis and pooling of results using Rubin’s rules would be computationally infeasible [14,20,21]. In addition to its practical advantages, average imputation has been shown to yield stable point estimates and is often used in exploratory analyses or when the focus is on estimating associations rather than fully propagating between-imputation uncertainty. Finally, modeling frameworks like BKMR that incorporate nonlinear, nonparametric structures often do not lend themselves easily to conventional pooling procedures like Rubin’s rules. In contrast, frequentist methods like WQS regression and quantile g-computation are better suited for Rubin’s rules due to their parametric estimates. For BKMR, averaging estimates across imputed datasets, while an approximation, offers a practical and increasingly standard approach in the field [22].

### 2.3. Bayesian Kernel Machine Regression

To assess the impact of pollutant mixtures on cardiovascular health, we implemented BKMR, a flexible, non-parametric approach suited for modeling complex, nonlinear relationships. BKMR effectively captures pollutant interactions and accounts for high-dimensional correlations prevalent in environmental datasets.

In the BKMR model specification, Y represent a health outcome, such as blood pressure or cholesterol levels, and X=(X₁,X₂,…,Xₙ) represents the mixture of environmental exposures. A hierarchical Bayesian framework is employed to estimate the function h(X). The BKMR model is defined as,

$$Y=h\left(X\right)+Z\beta +\epsilon$$

where h(X) is a flexible, non-parametric function modeled using a Gaussian process kernel. The Zβ represents covariates such as age, sex, BMI, and smoking status, while ε is the error term. Since directly modeling h(X) is complex, BKMR utilizes a kernel function to estimate h(X) more effectively. A hierarchical Bayesian framework is employed to evaluate both individual pollutant effects and their interactive influences. This approach enables posterior inference, allowing for the quantification of uncertainty and improving decision-making in environmental health research.

The function h(X) is modeled using a Gaussian Kernel, which provides a smooth and flexible estimation of the exposure–response relationship. The Gaussian Kernel is defined as,

(1) $$K\left(X_{i,}X_{J}\right)=\exp(-\sum_{m=1}^{M}\frac{{(X}_{im}-X_{jm}{)}^{2}}{\rho })$$

where

- $K(X_{i},X_{j})$ measures the similarity between exposure profiles of individuals i and j.
- $X_{im}$ and $X_{jm}$ represent the m-th pollutant exposure values for individuals i and j.
- M is the total number of pollutants in the mixture.
- ρ is a tuning parameter that controls the smoothness of the kernel function, where larger values result in smoother relationships, while smaller values capture more localized effects.

By incorporating the Gaussian Kernel into BKMR, the model effectively captures nonlinear exposure–response relationships, identifies interactions among pollutants, and enhances statistical inference in environmental health research.

### 2.4. Implementation of BKMR Modeling

The BKMR model was implemented to examine nonlinear exposure–response relationships and interactions between environmental pollutants. Prior to analysis, exposure variables were standardized via z-score normalization, ensuring comparability across pollutants. To account for potential confounding, covariates such as demographic characteristics were incorporated into the model.

The model estimation process followed a hierarchical Bayesian framework, using a Gaussian process kernel to capture complex dependencies. Metropolis–Hastings sampling was conducted with 5000 iterations to ensure the convergence of posterior distributions. Model selection and parameter tuning were optimized based on trace plots, posterior inclusion probabilities (PIPs), and overall risk summaries.

#### Evaluation and Interpretation of Results

Model diagnostics were assessed using trace plots to confirm Markov Chain Monte Carlo (MCMC) convergence. Predictor–response functions were generated to illustrate pollutant effects, while univariate response plots were utilized to detect interaction effects among chemical exposures.

PIPs were computed to rank the relative importance of individual pollutants. Exposure–response surfaces were analyzed to quantify the overall risk at different quantiles, allowing for a more precise evaluation of mixture effects. All statistical inferences were based on 95% credible intervals, ensuring the robustness of findings.

The computational implementation of BKMR provided a comprehensive framework for assessing high-dimensional environmental exposure data, offering insights into pollutant interactions and their cumulative effects on cardiovascular health.

Data analysis was conducted using R (version 4.2.3; R Foundation for Statistical Computing, Vienna, Austria). The following R libraries were used: Amelia for multiple imputation, bkmr for Bayesian Kernel Machine Regression, ggplot2 and gridExtra for visualization, and dplyr and tidyr for data wrangling. Python (version 3.9.12; Python Software Foundation, 2022) was used for generating customized visual plots and figure formatting.

## 3. Results

### 3.1. Descriptive Statistics

Table 1 summarizes the baseline sociodemographic characteristics and environmental exposure levels of participants from the 2013–2014 cycle of the NHANES. Demographic variables include age, body mass index (BMI), gender, race/ethnicity, education level, annual household income, smoking status, and alcohol consumption. The sample consists of over 10,000 individuals, with a near-even distribution by gender and diverse representation across racial and socioeconomic groups. Behavioral factors such as the history of smoking (≥100 cigarettes in lifetime) and alcohol use (≥12 drinks in the past year) are also captured.

The table further reports exposure concentrations for a range of environmental chemicals, including 13 heavy metals (e.g., lead, cadmium, mercury, arsenic, molybdenum) and 9 PFAS compounds (e.g., PFOA, PFHxS, PFNA), measured in the blood or urine. Additionally, 17 phthalates and plasticizers, assessed in the urine, are reported. Metal concentrations are expressed in micrograms per liter (µg/L), while PFAS, phthalate, and plasticizer levels are reported in nanograms per milliliter (ng/mL). Values are presented as means with standard deviations. These summary statistics provide a detailed overview of the study population’s exposure profiles and are used to explore associations with cardiovascular health outcomes in subsequent analyses.

### 3.2. Correlation Analysis

**Figure 1:**
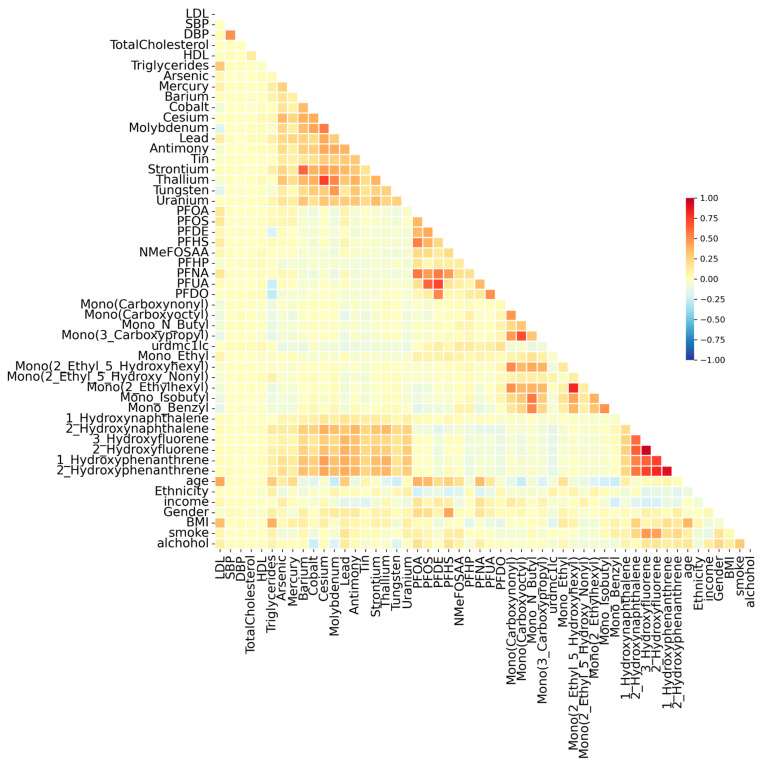
Pairwise associations between metabolic and cardiovascular biomarkers. Each heatmap represents the associations of different biomarker groups with various metabolites, with the strength and direction of associations indicated by color intensity (red: positive association; blue: negative association). The biomarkers include low-density lipoprotein (LDL), systolic blood pressure (SBP), diastolic blood pressure (DBP), total cholesterol, high-density lipoprotein (HDL), and triglycerides.

Pairwise Spearman correlation coefficients were computed to evaluate interrelationships between chemical exposures, covariates (e.g., age, BMI), and their associations with key cardiovascular health markers. The correlation matrix (Figure 1) reveals varying degrees of association among pollutants, with some clusters indicating shared environmental or metabolic sources. Stronger correlations were observed among certain metals, like cesium, barium, strontium, and thallium. PFAS compounds (e.g., PFUA, PFDO, PFHS, PFNA, PFDE) exhibited strong correlations, and some phthalates and plasticizers (e.g., 2-hydroxynaphthalene, 1-hydroxyphenanthrene, 2-hydroxyphenanthrene) demonstrated moderate interrelationships with some metals (e.g., lead, cesium, antimony), reflecting their frequent co-occurrence in plastics, food packaging, and personal care products. The correlation matrix reveals moderate associations between triglycerides and specific pollutants, PFDO, PFUA, and PFDE. In addition to pollutant interactions, several covariates showed strong correlations with cardiovascular markers. BMI was moderately correlated with LDL and triglycerides, with others being weak. Age displayed a strong positive association with SBP. The correlation structure informed subsequent mixture modeling in BKMR, ensuring that highly collinear variables were accounted for, to prevent statistical distortions and enhance interpretability to identifying key exposures.

### 3.3. Bayesian Kernel Machine Regression

BKMR modeling revealed complex nonlinear relationships between pollutant exposures and cardiovascular risk markers. Certain pollutants displayed threshold effects, where health impacts became evident only beyond specific exposure levels. Additionally, interactive effects were observed, suggesting that co-exposures may amplify or mitigate adverse health outcomes.

BKMR modeling revealed distinct nonlinear and interactive effects between environmental exposures and cardiovascular biomarkers. For example, PFOS and tungsten showed positive, monotonic associations with LDL, while PFDO was linked to increased triglyceride levels primarily above the median exposure level. Metals such as thallium and cesium demonstrated threshold effects on SBP, and phthalates like MEHHP exhibited inverse relationships with HDL. These results also suggested potential synergistic effects among certain pollutant groups, reinforcing the need for mixture-based evaluations in cardiovascular risk assessment.

#### Posterior Inclusion Probabilities (PIPs)

**Figure 2:**
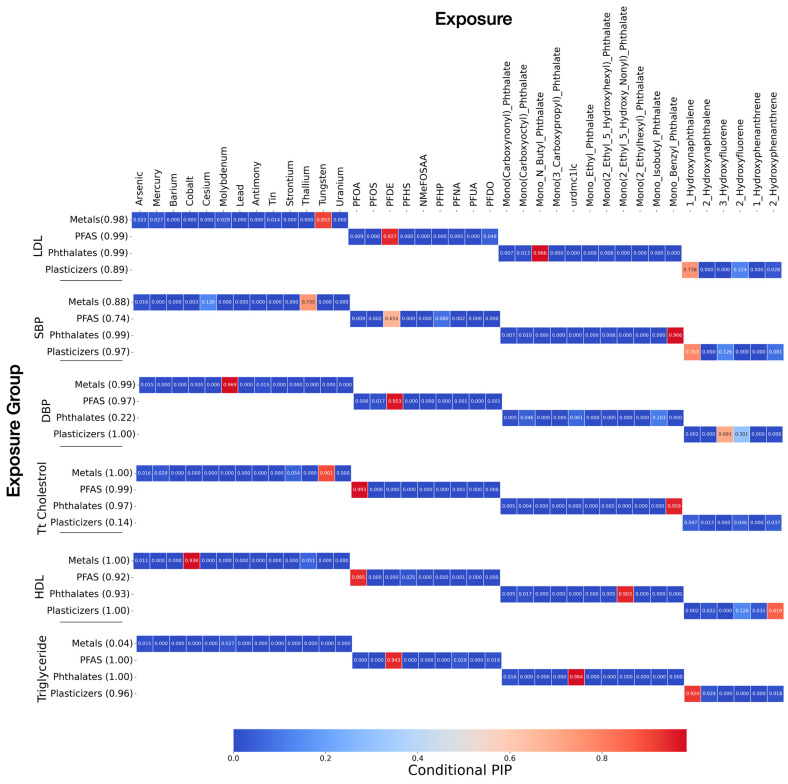
Group and conditional posterior inclusion probabilities (PIPs) for associations between environmental exposures and cardiovascular biomarkers. Each row represents a biomarker—LDL, SBP, DBP, total cholesterol, HDL, and triglycerides—while columns represent specific environmental exposures, grouped by chemical class (metals, PFAS, phthalates, plasticizers). Group PIPs (in parentheses) reflect the overall importance of each chemical group. Conditional PIPs, visualized by color intensity (red = higher inclusion probability), indicate the marginal influence of individual exposures within their groups.

To identify the most influential pollutants, we computed posterior inclusion probabilities (PIPs) (Figure 2). In the BKMR framework, the PIP reflects the likelihood that a specific exposure is meaningfully associated with the outcome, after accounting for all other variables. A PIP value close to 1 indicates strong evidence that the exposure contributes to the health effect, while values near 0 suggest little or no contribution.

The conditional PIP identifies the most influential pollutant within the group. The group and conditional PIP results show the most influential exposure groups and specific pollutants contributing to cardiovascular health outcomes. Metals, PFAS, and plasticizers had high summed group PIPs, indicating their strong impact across multiple health markers. Metals were associated with LDL, HDL, DBP, and total cholesterol, suggesting their role in lipid and blood pressure regulation. PFAS exposures showed a notable link to triglycerides, LDL, HDL, DBP, and total cholesterol. Phthalates had an influence on HDL, SBP, LDL, triglyceride, and total cholesterol, reinforcing concerns about their role in altering lipid profiles. Plasticizers had a high influence on DBP, SBP, triglyceride, and HDL.

The conditional PIP results further identify individual pollutants driving these associations. Hydroxynaphthalene, tungsten, PFDE, and monobutyl phthalate (MBP) were the strongest contributors to LDL, indicating potential disruptions in cholesterol regulation. DBP was primarily influenced by PFDE and molybdenum, suggesting a link between these pollutants and vascular dysfunction. For SBP, monobenzyl phthalate (MBzP) emerged as the most relevant exposures, pointing to potential hypertensive effects. PFOA, tungsten, and monobenzyl phthalate (MBzP) were strongly linked to total cholesterol, reinforcing their role in lipid metabolism. HDL was negatively associated with cobalt, PFOA, and mono(2-ethyl-5-hydroxyhexyl) phthalate (MEHHP), supporting evidence that certain phthalates and PFAS may lower protective cholesterol levels [23,24]. Lastly, triglyceride levels were most affected by PFDE and 1-hydroxynaphthalene, highlighting its potential metabolic impact.

It is important to note that in cases where pollutants within a class are highly correlated, BKMR often assigns high conditional PIP values to only one or a few representative chemicals. This reflects the model’s use of sparsity-inducing priors, which tend to select variables with the greatest marginal contribution while down-weighting those with overlapping information. As such, low PIPs for correlated variables do not necessarily imply lack of biological relevance but may instead reflect statistical redundancy within the model framework [14].

**Figure 3:**
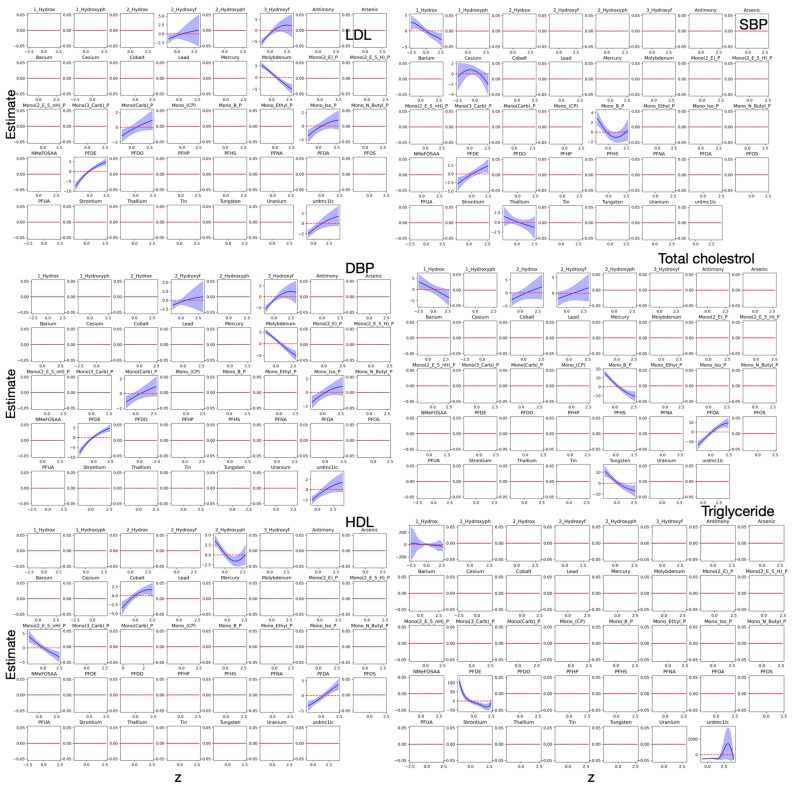
Univariate exposure–response functions examining the associations between individual pollutants and six key cardiovascular biomarkers. Each panel plots standardized exposure levels on the *x*-axis against the estimated effect on a specific biomarker on the *y*-axis, with shaded blue regions representing 95% credible intervals that reflect the uncertainty in the estimates.

Figure 3 shows the univariate exposure–response functions estimated from Bayesian Kernel Machine Regression (BKMR), illustrating the marginal associations between individual environmental pollutants and a set of cardiovascular biomarkers. In each plot, the estimated effect of a single pollutant on an outcome is shown while holding all other exposures constant at their median values. The results reveal a mix of linear and nonlinear associations, including several threshold and non-monotonic effects. These nonlinearities may reflect biological compensatory mechanisms, metabolic saturation, or endocrine-disrupting properties. Notably, inverse associations observed for some metals and phthalates should not be interpreted as protective. These patterns may reflect true biological mechanisms such as oxidative stress responses, metabolic compensation, or competitive absorption as described in the toxicological literature. However, they may also result from statistical conditioning within the model, particularly in the presence of highly correlated exposures. BKMR addresses such complexities through its hierarchical Bayesian structure and kernel-based estimation, which allow for flexible modeling of nonlinear and joint exposure effects. The model estimates conditional associations by holding other exposures constant, which can produce differing effect directions even within the same chemical class. These findings highlight the need for further mechanistic and experimental research to clarify the underlying drivers of these associations [25,26,27,28].

For LDL, higher levels are associated with increasing exposure to several phthalate metabolites such as mono(2-ethyl-5-carboxypentyl) phthalate, mono(2-ethylhexyl) phthalate, and mono(isobutyl) phthalate, as well as PFDE. These associations appear linear or near-linear, suggesting a consistent adverse lipid-altering effect across the exposure range. A distinct U-shaped pattern is observed for 3-hydroxyphthalate, with LDL levels peaking at moderate exposure levels, potentially reflecting threshold-dependent metabolic disruption. Conversely, molybdenum exhibits an inverse association with LDL, which may imply altered metal homeostasis. Other exposures, including PFNA and several plasticizers, show no appreciable association, as indicated by flat curves with narrow uncertainty bands.

SBP exhibits predominantly nonlinear responses. Cesium shows a concave association, while monobenzyl phthalate displays a convex pattern, both of which suggest complex dose–response dynamics. Interestingly, 1-hydroxyphthalate and thallium show steep declines in SBP at low to moderate concentrations, potentially indicative of competitive inhibition. PFDE presents a strong positive relationship with SBP, mirroring its effect on LDL and reinforcing its potential role in cardiometabolic dysregulation. Meanwhile, exposures such as strontium and barium show no significant associations, suggesting limited impact within the exposure ranges observed.

DBP is positively associated with multiple exposures, including 2-hydroxyphthalate, 3-hydroxyphthalate, PFDE, and various phthalates such as mono(carboxypropyl) phthalate and mono(isobutyl) phthalate. These associations demonstrate steepening exposure–response curves across the distribution, pointing to cumulative hypertensive effects of these ubiquitous toxicants. This supports emerging evidence that phthalates and PFAS may interfere with vascular function, renal signaling, or neuroendocrine regulation of blood pressure [23].

For total cholesterol, positive associations are observed with 2-hydroxynaphthalene, 2-hydroxyphthalate, and PFOA, all showing rising cholesterol levels with increasing exposure. In contrast, inverse associations are seen with 1-hydroxynaphthalene, monobenzyl phthalate, and tungsten, highlighting the heterogeneous effects of pollutants on lipid metabolism. Triglyceride levels appear most sensitive to monobenzyl phthalate and PFDE, both of which show declining trends at higher exposure levels. HDL, a protective biomarker, exhibits inverse associations with mono(2-ethyl-5-hydroxyhexyl) phthalate and 2-hydroxyphthalate, while positive associations are noted for cobalt and PFOA. These opposing effects on HDL may reflect underlying oxidative pathways triggered by different classes of toxicants.

Overall, the diverse response shapes—ranging from monotonic to curvilinear and null—highlight the necessity of flexible, nonparametric modeling approaches for capturing the nuanced effects of environmental exposures on cardiometabolic health.

**Figure 4:**
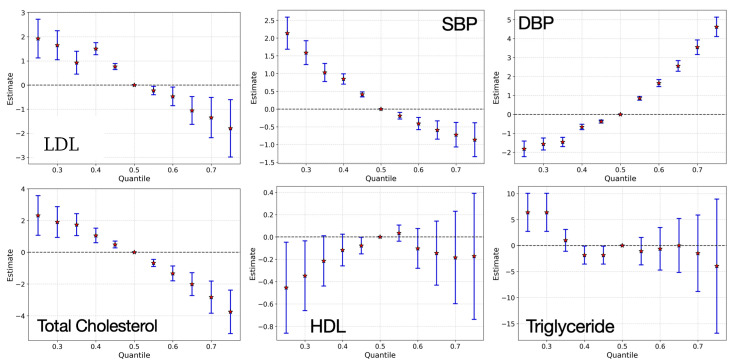
Overall exposure effects of all pollutants on CVD markers from the 0.25 quantile to the 0.75 quantile as compared to the 0.5 quantile. Each panel represents a specific biomarker, including LDL, SBP, DBP, total cholesterol, HDL, and triglycerides. The red dot is the estimate, and the blue lines are the 95% credible intervals.

Figure 4 shows the overall exposure effect of all the pollutants on individual cardiovascular markers. Higher exposure levels were linked to lower LDL and total cholesterol, showing a strong downward trend beyond the 50th quantile, suggesting potential disruptions in lipid metabolism. DBP increased with exposure, indicating a possible link between pollutants and higher blood pressure. SBP showed a downward trend but with minimal variability also indicating a disruption, suggesting a more complex relationship. Triglycerides and HDL showed a complex trend with significant variability at different exposure levels, an unexpected pattern that may point to underlying physiological responses.

To estimate these overall effects, we evaluated the predicted change in each cardiovascular marker when all exposures in the mixture were jointly set to specific quantiles of their distributions. Specifically, predictions were generated when all exposures were fixed at the 25th through 75th percentiles and compared to a reference scenario where all exposures were held at the median (50th percentile), while covariates were held constant.

## 4. Discussion

This study provides important insights into the health implications of pollutant mixtures, particularly metals, PFAS, phthalates, plasticizers, and their associations with CVD risk. The findings highlight the complex and multifactorial nature of environmental chemical exposures, which rarely occur in isolation. Instead, individuals are continuously exposed to combinations of pollutants through various pathways, including dietary intake, occupational settings, contaminated drinking water, and consumer products. The observed associations between these mixtures and cardiovascular biomarkers highlight how these exposures, acting alone or synergistically, may disrupt biological processes related to lipid metabolism, blood pressure regulation, and vascular function [25,26,29].

When further exploring the structure of these exposures, correlation analysis revealed clear groupings among pollutant classes. Strong correlations among PFAS compounds such as PFOS, PFNA, and PFDO are consistent with prior studies, indicating that these chemicals frequently co-occur in industrial and household products. Similarly, metals like cesium, barium, strontium, and thallium showed strong intercorrelations, likely reflecting their shared presence in environmental contamination and cumulative exposure over time. These patterns reflect the findings from Zanobetti, A., et al. (2014) and Gao, H., et al. (2011) [30,31], who noted pollutant clusters in large biomonitoring datasets. Some phthalates and plasticizers exhibited moderate interrelationships with some metals, which also aligns with previous evidence that these compounds are often encountered together in personal care products, plastics, and food packaging [32]. The correlations between pollutants and key cardiovascular biomarkers further support the potential for adverse health effects.

BKMR further illuminated these complex relationships by modeling nonlinear and interactive effects among pollutants. Certain chemicals demonstrated threshold effects, where adverse health outcomes only became apparent beyond specific exposure levels.

For instance, PFDE showed a gradual to sharp decrease in triglyceride levels after the 40th percentile of exposure, and PFDE was associated with elevated LDL cholesterol only at higher exposures. This is particularly relevant for endocrine-disrupting chemicals, where non-monotonic dose–response relationships have been widely reported [27]. Stronger correlations were observed among certain metals, like cesium, barium, strontium, and thallium. PFAS compounds (e.g., PFUA, PFDO, PFHS, PFNA, PFDE) exhibited strong correlations, and some phthalates and plasticizers (e.g., 2-hydroxynaphthalene, 1-hydroxyphenanthrene, 2-hydroxyphenanthrene) demonstrated moderate interrelationships with some metals (e.g., lead, cesium, antimony), reflecting their frequent co-occurrence in plastics, food packaging, and personal care products. The correlation matrix reveals moderate associations between triglycerides and specific pollutants, PFDO, PFUA, and PFDE.

In addition to pollutant interactions, several covariates showed strong correlations with cardiovascular markers. BMI was moderately correlated with HDL, with others being weak. Age displayed a strong positive association with SBP, confirming previous research [5,6] that demonstrated metal-induced dysregulation of blood pressure and lipid metabolism. PFAS compounds were primarily linked to triglycerides, LDL, and total cholesterol, consistent with findings by [33], which associated PFAS exposure with cholesterol abnormalities and vascular dysfunction. Phthalates emerged as important contributors to altered HDL and LDL levels, with MBP and MBzP showing significant effects. These associations parallel the findings, which reported elevated blood pressure and dyslipidemia among individuals with higher phthalate exposure [34,35]. Plasticizers also influenced cardiovascular outcomes, particularly in relation to diastolic blood pressure and LDL cholesterol [36]. The univariate exposure–response plots supported the presence of nonlinear associations and identified individual pollutants with strong, specific impacts on CVD biomarkers.

The PIP analysis further distinguished the most influential exposures, offering a ranked assessment of both individual chemicals and chemical classes. Notably, PFDE, urdmc1lc, and 1-hydroxynaphthalen were linked to triglycerides, tungsten, and PFDE, and monobenzyl phthalate was linked to total cholesterol. Exposure variables such as PFDE and tungsten contributed to increased LDL levels. Molybdenum and PFDE were associated with elevated DBP, while tungsten and monobenzyl phthalate were strongly tied to increased SBP. These findings align with the growing body of literature that emphasizes not just the presence of these pollutants in the environment but their potential to interfere with cardiovascular physiology through oxidative stress, inflammation, and endocrine disruption [28,37,38].

The application of BKMR enabled a more nuanced evaluation of how pollutant classes interact to influence cardiovascular outcomes. In contrast to traditional linear models, which may overlook synergistic effects or nonlinearity, BKMR captures both joint and individual contributions of exposures, revealing more accurate representations of real-world exposures [14]. This analytic approach aligns with recent calls in the literature to move beyond single-pollutant models and adopt frameworks that reflect the complexity of environmental exposures. This study affirms that single-chemical assessments miss the complexity of real-world exposures, as per the findings from Braun, J.M., et al. (2016) and Carlin, D.J., et al. (2013) [2,39], and our results support mixture-based approaches to better assess health risks. Using BKMR, we identified key pollutants affecting cardiovascular biomarkers and captured nonlinear, interactive effects overlooked by traditional models.

BKMR effectively addresses high-dimensional exposures and collinearity, making it ideal for complex chemical interactions [14,40,41,42]. We observed associations between PFAS, metals, phthalates, and various cardiovascular biomarkers that aligned with the findings from other studies [7]. These findings emphasize the need for cumulative, class-based regulation over single-chemical policies and highlight the potential of integrating BKMR to advance precision environmental health by identifying high-risk groups and informing targeted interventions [43].

However, some limitations must be acknowledged. While NHANES provides high-quality, nationally representative data, certain methodological considerations are important to acknowledge. First, the dataset is cross-sectional, which limits causal interpretation. Second, environmental biomarkers are measured in a randomly selected subsample (~25% of the full NHANES cohort) as part of NHANES’s complex multistage probability sampling design. This subsampling is not based on exposure or outcome status and is designed to preserve national representativeness.

To address missing data in exposure and covariate variables, we applied multiple imputation using a robust expectation-maximization with bootstrapping (EMB) algorithm, incorporating a broad set of auxiliary variables to support the Missing at Random (MAR) assumption. Estimates were combined across ten imputed datasets, ensuring valid inference while retaining a large, diverse analytic sample. These methodological steps strengthen the robustness of our findings and support the reliability of the exposure–response relationships observed.

To build on this work, future research should prioritize longitudinal designs capable of assessing cumulative exposures over time and tracking changes in health outcomes.

Future work should expand the application of BKMR to include broader health indicators, such as inflammatory and metabolic biomarkers, which will allow for a more comprehensive understanding of systemic impacts. Integrating BKMR with other machine learning techniques may also improve model sensitivity to complex interactions and allow for better prediction of high-risk subgroups. Nevertheless, despite these limitations, this study provides important insights into the potential health effects of complex chemical mixtures. The use of BKMR allowed for the identification of nonlinear associations and interactions that may be missed by conventional models, offering a more realistic representation of environmental exposures. The observed patterns highlight key pollutants and mixture effects that warrant further investigation and may guide future research and policy efforts to reduce population-level health risks.

## 5. Conclusions

This study shows how complex chemical mixtures can affect cardiovascular health. By using BKMR, we captured how these pollutants interact and influence health outcomes in ways that single-chemical analyses may miss. Current regulations often assess chemicals individually, which can underestimate real-world risks. Our findings highlight the importance of evaluating pollutant mixtures. BKMR provides a more accurate and realistic approach to understanding these combined effects, supporting the need for mixture-based risk assessment frameworks. Given the widespread presence of these chemicals in industrial emissions, consumer products, and food packaging, there is a need for class-based regulation and cumulative exposure policies.

As regulatory agencies increasingly recognize the limitations of single-chemical assessments, the integration of mixture modeling frameworks like BKMR could enhance toxicological risk evaluations by accommodating real-world exposure complexity. This aligns with guidance from the U.S. Environmental Protection Agency (EPA) on cumulative risk assessment, as well as recommendations from the National Academies of Sciences to improve chemical evaluation methods in the face of environmental mixture exposures. Wider adoption of these approaches—supported by open-source tools, cross-disciplinary collaboration, and accessible training—could strengthen regulatory science and promote more protective, evidence-based environmental health policies.

## Figures and Tables

**Table 1:** Baseline characteristics and exposure levels of study participants.

Variable	n	Mean (SD)
Age (years)	10,175	31 (24)
BMI (kg/m^2^)	9055	25.68 (7.96)
		**Percentage (%)**
Alcohol (Had at least 12 alcohol drinks/1 yr?)		
Yes	3790	37.25
No	1623	15.95
Don’t know	8	0.08
Smoke (Smoked at least 100 cigarettes in life)		
Yes	2579	25.35
No	3532	34.71
Don’t know	2	0.02
Education (Education level—Adults 20+)		
Less than 9th grade	455	4.47
9–11th grades (Includes 12th grade with no diploma)	791	7.77
High school graduate/GED or equivalent	1303	12.81
Some college or AA degree	1770	17.40
College graduate or above	1443	14.18
Refused	2	0.02
Don’t know	5	0.05
Ethnicity (Race/Hispanic origin w/NH Asian)		
Mexican American	1730	17
Other Hispanic	960	9.43
Non-Hispanic white	3674	36.11
Non-Hispanic Black	2267	22.28
Non-Hispanic Asian	1074	10.56
Other Race—Including multi-racial	470	4.62
Gender		
Male	5003	50.83
Female	5172	49.17
Income (USD): Annual household income		
USD 0 to USD 4999	273	2.46
USD 5000 to USD 9999	407	4
USD 10,000 to USD 14,999	639	6.28
USD 15,000 to USD 19,999	658	6.47
USD 20,000 to USD 24,999	880	8.65
USD 25,000 to USD 34,999	1185	11.65
USD 35,000 to USD 44,999	913	8.97
USD 45,000 to USD 54,999	764	7.51
USD 55,000 to USD 64,999	521	5.12
USD 65,000 to USD 74,999	378	3.71
USD 20,000 and over	323	3.17
Under USD 20,000	133	1.31
USD 75,000 to USD 99,999	860	8.45
USD 100,000 and over	1781	17.50
Refused	252	2.48
Don’t know	75	0.74
**Metals**		
Lead (Pb, µg/dL)	2664	0.46 (0.77)
Cadmium (Cd, µg/L)	2664	0.24 (0.36)
Thallium (TI, µg/L)	2664	0.19 (0.13)
Molybdenum (Mo, µg/L)	2664	56.10 (56.12)
Arsenic (As, µg/L)	2662	15.67 (46.74)
Mercury (Hg, µg/L)	2666	0.51 (2.04)
Barium (Ba, µg/L)	2664	1.77 (3.00)
Cobalt (Co, µg/L)	2664	0.62 (1.78)
Cesium (Cs, µg/L)	2664	4.97 (3.14)
Manganese (Mn, µg/L)	2664	0.16 (0.49)
Antimony (Sb, µg/L)	2664	0.08 (0.14)
Tin (Sn, µg/L)	2664	1.44 (4.71)
Strontium (Sr, µg/L)	2664	118.07 (182.79)
Tungsten (W, µg/L)	2664	0.15 (0.58)
Uranium (U, µg/L)	2664	0.01 (0.03)
**PFAS**		
PFOA (ng/mL)	1954	2.33 (3.01)
PFOS (ng/mL)	1954	8.02 (32.70)
PFHS (ng/mL)	2168	1.94 (2.21)
PFDE (ng/mL)	2168	0.31 (1.21)
NMeFOSAA (ng/mL)	2168	0.18 (0.29)
PFHP (ng/mL)	2168	0.08(0.06)
PFNA (ng/mL)	2168	0.87 (0.83)
PFUA (ng/mL)	2168	0.23(1.93)
PFDO (ng/mL)	2168	0.10 (0.16)
**Phthalates and Plasticizers**		
Mono(carboxynonyl)_phthalate (ng/mL)	2685	5.99 (25.40)
Mono(carboxyoctyl)_phthalate (ng/mL)	2685	54.09 (115.83)
Mono(2-ethyl-5-carboxypentyl)_phthalate (ng/mL)	2685	20.92(44.24)
Mono-n-butyl_phthalate (ng/mL)	2685	17.93 (25.84)
Mono(3-carboxypropyl)_phthalate (ng/mL)	2685	5.31 (15.45)
urdmc1lc (ng/mL)	2685	0.10 (0.30)
Mono-ethyl_phthalate (ng/mL)	2685	200.85(1067.64)
Mono(2-ethyl-5-hydroxyhexyl)_phthalate (ng/mL)	2685	13.64 (32.82)
Mono(2-ethyl-5-hydroxy-nonyl)_phthalate (ng/mL)	2685	0.72 (3.40)
Mono(2-ethylhexyl)_phthalate (ng/mL)	2685	2.45 (5.33)
Mono-isobutyl_phthalate (ng/mL)	2685	14.77 (23.48)
Monobenzyl phthalate (ng/mL)	2685	11.24 (22.46)
1-Hydroxynaphthalene (ng/L)	2640	27,874.13 (724,051.50)
2-Hydroxynaphthalene (ng/L)	2641	8737.88 (13,875.15)
3-Hydroxyfluorene (ng/L)	2650	218.96 (462.62)
2-Hydroxyfluorene (ng/L)	2650	390.08 (713.54)
1-Hydroxyphenanthrene (ng/L)	2650	142.18 (223.26)
2-Hydroxyphenanthrene (ng/L)	2650	188.78 (293.77)

## Data Availability

The NHANES dataset is publicly available online, accessible at https://www.cdc.gov/nchs/nhanes/ (accessed on 4 April 2025).
